# Sepsis-associated endothelial dysfunction: clinical phenotypes, biomarkers, bedside evidence, and translational implications

**DOI:** 10.1172/JCI208809

**Published:** 2026-10-15

**Authors:** Song Qin, Ninan Dai, Fengyun Xu, Cindy Zhuang, Judith Hellman

**Affiliations:** 1 Department of Anesthesia and Perioperative Care, UCSF School of Medicine, San Francisco, California, USA.; 2 Department of Critical Care Medicine, Affiliated Hospital of Zunyi Medical University, Zunyi, Guizhou, China.; 3 SUNY Upstate Medical University, Syracuse, New York, USA.

## Abstract

Sepsis is a systemic vascular disorder in which endothelial activation, dysfunction, and structural injury contribute to shock, microcirculatory failure, immunothrombosis, and multiorgan dysfunction. Sepsis-associated endotheliopathy is not a uniform process but a dynamic trajectory that evolves from adaptive endothelial activation toward functional disruption, structural injury, and organ-specific vascular failure. In this Review, we propose a phenotype- and trajectory-oriented framework that integrates clinical manifestations, molecular mechanisms, and translational assessment of endothelial dysfunction in sepsis. We focus on four major clinically recognizable modes of endothelial dysfunction that often overlap and evolve over the course of sepsis. We further discuss biology-anchored biomarker panels, bedside physiological surrogates, and serial trajectory-based assessment as complementary approaches for identifying endothelial states, refining risk stratification, and guiding future precision strategies. Finally, we describe implications for biomarker-enriched clinical trials, microvascular-informed resuscitation, and endotype-guided endothelial cell–targeted therapies. Viewing sepsis through the lens of dynamic endotheliopathy provides a framework linking vascular biology to clinically interpretable trajectories and may facilitate the development of personalized approaches to endothelial cell–directed care.

## Introduction

Sepsis, an extreme response to infection, remains a major global health challenge, causing high mortality rates and long-term health complications. There is considerable variability in sepsis incidence, prevalence, and mortality across regions and healthcare systems (1). This clinical variability complicates risk assessment and precise treatment (2). The heterogeneity in sepsis is also reflected in diverse vascular manifestations observed at the bedside. Some patients have capillary leakage and tissue edema, others demonstrate persistent tissue hypoperfusion despite restoration of systemic hemodynamics, and still others develop prominent coagulation abnormalities, microvascular thrombotic events, or inflammatory organ injury. These patterns frequently overlap and may evolve dynamically over the course of illness, highlighting the complexity of sepsis-associated vascular dysfunction.

The adult human body is estimated to contain approximately 750 billion endothelial cells distributed across all vascular beds (3). The endothelium is essential for protective host responses to infection and injury, acting by maintaining barrier integrity and hemostasis, regulating microvascular tone, guiding leukocyte movement between intravascular and extravascular spaces, and supporting metabolic signaling (4). However, sepsis profoundly affects vascular and endothelial cell functions (4). Sepsis-induced endothelial dysfunction, also referred to as sepsis-associated endotheliopathy (SAE), can result in capillary leak, vasoplegia (5, 6), microcirculatory hypoperfusion (7, 8), immunothrombosis (9, 10), and multiorgan dysfunction (5).

Current evidence supports a dynamic transition from early endothelial activation, which can facilitate host defense, toward progressive endothelial dysfunction, structural injury, and the development of overt organ failure (11). Within this continuum, four dominant manifestations can emerge: barrier disruption with capillary leak; vasoregulatory and microcirculatory dysfunction; proadhesive/procoagulant activation with immunothrombosis; and leukocyte adhesion–driven inflammatory amplification (Figure 1). These manifestations represent overlapping consequences of shared endothelial injury programs, with their relative contributions varying according to disease stage, inflammatory burden, therapeutic interventions, and the structural and functional characteristics of individual vascular beds. Consequently, endothelial dysfunction is not expressed uniformly across organs but instead gives rise to distinct patterns of tissue vulnerability and organ-specific injury (Figure 2).

Here, we present a phenotype- and trajectory-oriented framework to organize SAE into clinically recognizable modes of endothelial failure that evolve dynamically over the course of sepsis. This framework links molecular mechanisms, bedside manifestations, and longitudinal biological and clinical changes. Our goal is to provide a structure for identifying the predominant endothelial state at a given time point, integrating biomarkers with physiological surrogates, and understanding trajectories of recovery or progression to organ injury and failure (Figure 3 and Table 1).

## Clinical manifestations of sepsis-induced endothelial dysfunction

SAE manifests as four dynamic and overlapping modes of endothelial failure: disturbances in vascular barrier integrity with hyperpermeability; vasoregulatory dysfunction with microvascular hypoperfusion; hemostatic regulation; and inflammatory cell trafficking. There may be a dominant disturbance that most strongly shapes an individual patient’s physiological and organ-level presentation at a given time, or multiple disturbances can coexist, leading to complex pathophysiology. The relative contributions of different modes of endothelial dysfunction vary according to disease stage, inflammatory burden, vascular bed susceptibility, and therapeutic interventions. The endothelial dysfunction modes lead to diverse clinical manifestations, such as hypovolemia, impaired vascular tone, microvascular hypoperfusion, shock, and systemic and organ inflammation and injury, which can be assessed using biomarkers and functional surrogates.

Barrier failure and capillary leak are the clearly recognizable manifestations of SAE. Clinically, barrier dysfunction is suggested when progressive tissue edema and impaired fluid tolerance occur despite appropriate resuscitation. Patients may develop increasing tissue swelling, fluid accumulation, and worsening organ function, particularly when vascular leakage exceeds the capacity for fluid redistribution and clearance (4, 6, 12). The lung is particularly vulnerable to barrier disruption because of its extensive microvascular network and specialized alveolar-capillary interface. Pulmonary endothelial injury promotes protein-rich edema formation, impairs lung compliance, causes ventilation-perfusion mismatch, and contributes to progression toward acute respiratory distress syndrome (ARDS) (13). Similarly, intestinal vascular barrier disruption may cause bowel wall edema, impaired gastrointestinal motility, and mucosal barrier dysfunction, potentially amplifying systemic inflammation (14, 15). Renal endothelial injury may also interact with altered venous congestion and impaired microvascular exchange, contributing to the development of sepsis-associated acute kidney injury (AKI) (16, 17). Thus, barrier-dominant manifestations represent a clinically recognizable expression of endothelial dysfunction (18, 19).

A second major manifestation of SAE is impaired regulation of microvascular perfusion. Clinically, this may present as prolonged capillary refill time, skin mottling, persistent hyperlactatemia, or delayed lactate clearance despite adequate blood pressure and cardiac output (20–22). This mode is characterized by persistent evidence of tissue hypoperfusion even after apparent normalization of systemic hemodynamic parameters, reflecting a loss of coherence between the macro- and microcirculations. These findings suggest impaired distribution of microvascular blood flow and inadequate tissue oxygen utilization rather than insufficient systemic perfusion alone (23, 24). The persistence of microcirculatory abnormalities despite achieving conventional hemodynamic targets highlights the importance of endothelial regulation of capillary flow. However, these bedside indicators are not specific for endothelial dysfunction and should be interpreted in the context of metabolic status, organ function, and other potential contributors to impaired tissue oxygen balance.

SAE is also characterized by disruption of the physiological balance between anticoagulant and procoagulant mechanisms, leading to proadhesive/procoagulant activation and thromboinflammatory manifestations. Endothelial activation may initially support host defense by promoting localized immunothrombosis, limiting pathogen dissemination (9). However, excessive or persistent activation can result in pathological microvascular thrombosis and impaired tissue perfusion (25). Clinically, this presents as progressive thrombocytopenia, coagulation abnormalities, and organ dysfunction disproportionate to the systemic hemodynamic changes and results from microvascular obstruction and thromboinflammatory injury (26–29). These changes range in severity from sepsis-induced coagulopathy to overt disseminated intravascular coagulation, accompanied by progressive consumption of platelets and coagulation factors (30, 31). Notably, routine coagulation abnormalities alone may not directly demonstrate endothelial cell–driven microvascular thrombosis. A thromboinflammatory-dominant state is more strongly indicated when dynamic platelet reductions, endothelial coagulation biomarkers, and ischemic organ manifestations evolve concordantly (26–28, 32). Therefore, this manifestation reflects an imbalance between protective immune defense and pathological vascular thrombosis rather than coagulation activation alone.

Endothelial cells also actively contribute to localized and systemic inflammation and contribute to persistent inflammatory amplification by producing cytokines and chemokines and facilitating interactions between endothelial cells and circulating immune cells. Activated endothelial surfaces promote leukocyte adhesion, trafficking, and tissue infiltration, thereby sustaining organ-specific inflammatory injury (18, 33, 34). This process contributes to multiple organ manifestations of sepsis, including pulmonary inflammatory injury in ARDS (35), neurovascular dysfunction and neuroinflammation associated with sepsis-associated encephalopathy (36), and inflammation-driven sepsis-associated AKI (37). Inflammatory amplification frequently overlaps with barrier dysfunction and thromboinflammatory activation, reflecting shared upstream endothelial signaling pathways (38). In contrast to the other modes of endothelial dysfunction, inflammatory amplification lacks a specific bedside signature. It is primarily recognized through the combination of organ-directed inflammatory injury patterns and evidence of endothelial activation. Levels of soluble adhesion molecules may provide biological insight but require cautious interpretation owing to limited organ specificity, variable release kinetics, and dependence on disease stage (39, 40).

Collectively, these clinical manifestations represent complementary expressions of SAE. Defining these clinically recognizable manifestations provides a framework for subsequent examination of the molecular programs that drive endothelial activation, dysfunction, and injury during sepsis.

## A trajectory-oriented framework of SAE

Endothelial activation in sepsis is a dynamic process rather than a static pathological state. Following exposure to various inflammatory stimuli, including pathogen-associated molecular patterns (PAMPs), damage-associated molecular patterns (DAMPs), and inflammatory mediators, as well as hemodynamic and metabolic stress, endothelial cells undergo an initial activation response that coordinates beneficial responses, including host defenses and vascular adaptations (18, 41). During this early phase, increased expression of adhesion molecules, transient inflammatory signaling, and modulation of vascular permeability may facilitate immune surveillance and pathogen clearance.

As sepsis progresses, there is a transition from endothelial activation to endothelial failure. Persistent inflammation and vascular stress may drive endothelial cells from an adaptive activated state toward progressive functional impairment and, in severe cases, structural injury and organ-specific dysfunction. This manifests as disruption of key endothelial functions, including maintenance of the vascular barrier, regulation of microvascular perfusion, control of leukocyte trafficking, and preservation of hemostatic balance (41). Associated clinical manifestations, such as hypotension, ARDS, coagulopathy, and sepsis-associated encephalopathy, may emerge and be amplified by inflammation. Notably, these diverse manifestations of endotheliopathy arise from interconnected molecular programs (8, 12). With sustained endothelial stress, reversible functional alterations may progress to structural endothelial injury, characterized by loss of endothelial integrity, persistent microvascular dysfunction, impaired capacity for vascular repair, and disrupted tissue homeostasis (11).

Because endothelial cells exhibit organ-specific specialization, the clinical manifestations of sepsis-induced endotheliopathy vary across organs. The pulmonary circulation may predominantly manifest barrier failure, whereas renal, cerebral, and cardiovascular vascular beds may exhibit distinct patterns of microvascular dysfunction, impaired vascular regulation, and organ-specific injury (42). Therefore, determining an individual patient’s position along this evolving trajectory may provide clinically meaningful information to help guide longitudinal risk stratification.

The phenotype- and trajectory-oriented framework conceptualizes SAE as a dynamic process in which diverse patterns of endothelial dysfunction emerge and evolve over time. Their relative predominance at different stages of sepsis is determined by the intensity and duration of inflammatory stimulation, vascular bed susceptibility, host factors, and therapeutic interventions. Accordingly, endothelial states can be characterized across three dimensions: the predominant functional disturbance at a given time point, the temporal trajectory of disease evolution, and the relationship between biological alterations and physiological manifestations. Whereas early endothelial activation may represent an adaptive response that supports immune surveillance and vascular regulation, persistent endothelial stimulation can promote progressive barrier disruption, microcirculatory impairment, inflammatory amplification, and thromboinflammatory activation, and resolution of inflammatory stress and restoration of endothelial homeostasis may allow partial recovery of vascular function (18, 32, 43).

This dynamic model may explain why patients with similar clinical diagnoses of sepsis can exhibit heterogeneous vascular manifestations and different patterns of organ dysfunction. Rather than assigning patients to fixed categories, a trajectory-based interpretation emphasizes the evolving balance between endothelial injury and repair processes throughout the disease course. The integration of temporal changes with biological and clinical measurements provides a conceptual basis for further investigation of endothelial heterogeneity in sepsis and its potential implications for patient stratification and therapeutic development.

## Mechanisms of endothelial activation and dysfunction in sepsis

The endothelium is uniquely positioned to amplify inflammation and promote local microvascular injury despite hemodynamic stabilization (3–10). During sepsis, endothelial cells integrate signals from pathogens, injured tissues, immune cells, and circulating mediators, resulting in progressive alterations in barrier integrity, vascular tone, leukocyte trafficking, and coagulation balance. Innate immune system activation, cytokine signaling, complement activation, glycocalyx disruption, NO dysregulation, oxidative stress, and immunothrombosis each contribute to endothelial dysregulation in sepsis (Figure 1).

### Endothelial immune signaling in sepsis.

The endothelium functions as an active immune-responsive interface that integrates microbial signals, host-derived danger signals, and inflammatory cues. Endothelial cells recognize PAMPs and DAMPs through pattern recognition receptors (PRRs), thereby initiating programs that regulate vascular permeability, leukocyte trafficking, coagulation, and microvascular function (44).

Microbial components and tissue-derived danger signals activate endothelial PRRs, such as the TLRs (45). The engagement of endothelial innate immune receptors by microbial factors, such as lipopolysaccharide (LPS; TLR4 agonist) or bacterial lipoproteins (TLR2 agonists), or engagement of receptors for TNF-α, IL-1β, and other mediators, activates intracellular inflammatory pathways (46–48). This leads, via signaling through myeloid differentiation primary response 88 (MyD88) and/or TIR-domain-containing adapter-inducing interferon-β (TRIF), to activation of mitogen-activated protein kinases and activation and nuclear translocation of NF-κB, with downstream expression of cytokines and other inflammatory mediators and adhesion molecules, including ICAM-1, VCAM-1, and E-selectin (49–51).

Endogenous DAMPs, such as mitochondrial DNA and HMGB1, further amplify inflammatory signaling through TLRs and inflammasome-related pathways (38, 52, 53). The convergence of microbial and sterile inflammatory signals establishes a self-amplifying environment. Although these responses contribute to early host defense, excessive or persistent activation promotes endothelial dysfunction (54).

### Cytokine-mediated endothelial reprogramming in sepsis.

Cytokines represent a central communication network linking immune activation with endothelial reprogramming during sepsis. Following recognition of microbial components and tissue injury signals, activated immune cells release inflammatory mediators that act on endothelial cells to regulate permeability, leukocyte trafficking, vascular tone, and coagulation balance (55, 56). Activated endothelial cells themselves also produce cytokines and chemokines, creating a reciprocal signaling loop between immune cells and the vascular wall. Among circulating inflammatory mediators, TNF-α, IL-1β, and IL-6 are major regulators of endothelial dysfunction during sepsis (57). TNF-α and IL-1β activate TNFR- and IL-1R–dependent NF-κB and MAPK signaling pathways, inducing expression of adhesion molecules, inflammatory mediators, and procoagulant factors. Persistent cytokine stimulation promotes VE-cadherin redistribution, cytoskeletal remodeling, junctional instability, and impaired barrier regulation (58, 59). IL-6 further contributes to endothelial dysfunction through classical and trans-signaling pathways involving IL-6R and gp130, promoting inflammatory activation, vascular instability, tissue factor expression, and interactions among endothelial cells, platelets, and immune cells (60). Therefore, cytokine signaling represents a critical transition point linking early endothelial activation with persistent endothelial dysfunction, inflammatory amplification, and thromboinflammatory injury.

### Complement and the endothelium in sepsis.

Complement activation contributes to host defense by promoting pathogen clearance and immune recruitment, but excessive or sustained activation can become maladaptive, driving endothelial inflammatory activation and thromboinflammatory responses (61, 62). During sepsis, microbial components, immune complexes, and tissue injury signals activate complement, leading to the generation of C3a, C5a, and the membrane attack complex (MAC, C5b-9) (63, 64). Complement signaling promotes inflammatory gene expression, leukocyte recruitment, and endothelial activation (65). C5a-C5aR1 activation induces intracellular endothelial signaling responses, including activation of NF-κB and MAPK pathways, thereby amplifying inflammatory gene expression and endothelial stress responses (66, 67). In parallel, sublytic MAC formation activates endothelial cells via calcium signaling, oxidative stress generation, and release of inflammatory mediators, contributing to endothelial instability and impaired vascular regulation (68). Furthermore, activated complement signaling promotes endothelial expression of procoagulant mediators, including tissue factor, and enhances interactions among endothelial cells, platelets, and immune cells (69). C5a-induced neutrophil activation facilitates neutrophil extracellular trap (NET) formation, which provides a scaffold for platelet recruitment and coagulation activation while further amplifying complement signaling (70). Thus, complement activation represents a critical convergence point among inflammation, endothelial dysfunction, and immunothrombosis.

### The glycocalyx in sepsis.

The endothelial glycocalyx, a critical interface between circulating blood components and the endothelial surface, contributes to vascular barrier integrity, mechanotransduction, anticoagulant regulation, and inflammatory homeostasis (71). During sepsis, this highly organized structure undergoes profound disruption in response to inflammatory mediators, oxidative stress, enzymatic degradation, and hemodynamic disturbances; an early molecular event in the transition from endothelial activation to barrier dysfunction (72). Inflammatory cytokines, ROS, and activated leukocytes promote the release of glycocalyx-degrading enzymes, including heparanase, matrix metalloproteinases, and other proteases, leading to loss of glycocalyx components and disruption of endothelial surface integrity (73, 74). In addition, excessive complement activation and neutrophil-derived mediators further accelerate glycocalyx injury, amplifying endothelial activation and inflammatory cell recruitment (75).

Loss of glycocalyx integrity alters interactions between plasma components and the endothelial surface. Glycocalyx disruption also contributes to abnormal leukocyte adhesion and platelet-endothelial interactions by exposing endothelial adhesion molecules and reducing the protective anticoagulant properties of the endothelial surface (76, 77). These changes promote capillary leakage, tissue edema, microvascular inflammation, and impaired organ function.

Circulating glycocalyx-derived molecules, including components released during glycocalyx shedding, have been extensively investigated as potential indicators of endothelial injury in sepsis (11, 73, 74). However, these molecules primarily reflect downstream consequences of glycocalyx disruption and the extent of endothelial damage, rather than the initiating mechanisms responsible for barrier failure (11, 73, 74).

### Disruption of endothelial junctional structures in sepsis.

Sepsis-induced barrier failure is further mediated by the disruption of endothelial junctional structures, including adherens junctions and tight junctions. Inflammatory signaling pathways, cytoskeletal remodeling, and altered VE-cadherin organization weaken endothelial cell-cell contacts, resulting in sustained permeability abnormalities and loss of vascular homeostasis (78, 79). Of note, glycocalyx degradation and junctional disruption are interconnected processes that together determine the severity and reversibility of endothelial barrier dysfunction (73).

### NO dysregulation and microvascular dysfunction in sepsis.

NO is a central regulator of endothelial homeostasis, integrating the regulation of vascular tone, platelet activity, leukocyte-endothelial interactions, and microvascular perfusion. Under physiological conditions, NO derived from endothelial NO synthase maintains vascular function by promoting vasodilation, limiting platelet activation, and preserving endothelial responsiveness to changes in blood flow (80).

During the early phase of sepsis, transient activation of endothelial NO pathways may contribute to adaptive vascular responses by supporting tissue perfusion and modulating inflammatory interactions. However, as inflammation persists, NO production becomes dysregulated, shifting from tightly controlled endothelial signaling toward excessive, spatially heterogeneous NO generation. Proinflammatory cytokines, microbial products, and cellular stress signals promote expression of inducible NO synthase (iNOS) in immune and endothelial cells, resulting in sustained NO overproduction (81, 82). Excessive iNOS-derived NO contributes to vascular dysfunction through several mechanisms. Increased NO availability can reduce vascular smooth muscle responsiveness and impair vasoconstrictive mechanisms, contributing to septic vasoplegia and persistent hypotension despite adequate volume replacement and vasopressor therapy (83). In addition, excessive NO signaling alters endothelial regulation of microvascular blood flow, contributing to impaired capillary perfusion and loss of coherence between systemic hemodynamics and tissue oxygen delivery (84). Under inflammatory conditions, excessive NO interacts with ROS to generate reactive nitrogen species, further impairing endothelial signaling and vascular homeostasis (85, 86). Thus, NO dysregulation represents a key mechanism linking endothelial activation with vasoregulatory failure and microcirculatory abnormalities during sepsis.

### Oxidative stress and the endothelium in sepsis.

ROS are a common downstream mediator linking inflammatory activation, metabolic disturbance, and endothelial injury during sepsis. Under physiological conditions, endothelial cells maintain redox homeostasis through coordinated antioxidant systems that regulate cellular signaling and vascular function. During sepsis, excessive ROS production overwhelms antioxidant defenses, leading to oxidative stress and impaired endothelial homeostasis (87, 88). Multiple inflammatory pathways contribute to ROS accumulation during sepsis. Activation of immune cells, particularly neutrophils and macrophages, promotes respiratory burst responses and the generation of reactive intermediates, whereas mitochondrial dysfunction in endothelial cells further increases oxidative stress by impairing electron transport and altering cellular metabolism (89, 90). In addition, enzymes such as NADPH oxidases and uncoupled NO synthase contribute to sustained ROS production, thereby amplifying endothelial injury (91).

Oxidative stress affects endothelial function through multiple mechanisms. ROS-mediated modification of cellular proteins, lipids, and nucleic acids disrupts endothelial signaling, impairs barrier integrity, and promotes inflammatory activation (92). ROS also enhance leukocyte adhesion, platelet activation, and coagulation responses, thereby linking oxidative injury with thromboinflammatory processes (93). Furthermore, oxidative stress can exacerbate glycocalyx degradation and endothelial junctional disruption, increasing vascular permeability and tissue edema (94). Thus, oxidative stress is a shared amplification mechanism that intensifies multiple pathways of endothelial dysfunction. By reinforcing inflammatory signaling, barrier disruption, microvascular abnormalities, and immunothrombotic activation, oxidative stress contributes to the progression from reversible endothelial activation toward persistent vascular injury during sepsis.

### NETs in sepsis.

Activated neutrophils release NETs, which are extracellular DNA structures decorated with histones and antimicrobial proteins. NETs serve as a scaffold for platelet adhesion, coagulation activation, and endothelial injury (95, 96). NETs are believed to initially contribute to pathogen containment, but excessive NET accumulation promotes persistent endothelial activation, vascular occlusion, and amplification of inflammatory injury (95, 96).

### Endothelial immunothrombosis.

Immunothrombosis represents a critical transition of the endothelium from an anticoagulant and homeostatic surface toward a procoagulant and inflammatory state. Under physiological conditions, endothelial cells maintain vascular fluidity through anticoagulant mechanisms, including the expression of thrombomodulin and the endothelial protein C receptor (EPCR), as well as the regulation of platelet-endothelial interactions (25). During sepsis, persistent inflammatory stimulation disrupts these protective pathways, leading to enhanced coagulation, platelet recruitment, and microvascular thrombus formation (9, 25).

A central event in endothelial immunothrombosis is the induction of a procoagulant endothelial phenotype. Inflammatory cytokines, microbial products, and activated immune cells promote endothelial expression of tissue factor, initiating thrombin generation and fibrin deposition within the microcirculation (97, 98). Concurrently, downregulation of anticoagulant pathways, including impaired thrombomodulin and EPCR signaling, further shifts the vascular environment toward coagulation activation and loss of endothelial homeostasis (99, 100).

The vWF a disintegrin and metalloprotease with thrombospondin type 1 motif 13 (ADAMTS13) axis is another important regulator of endothelial thromboinflammatory responses. Activated endothelial cells release large amounts of vWF to promote platelet adhesion; however, reduced ADAMTS13 activity during sepsis limits cleavage of ultralarge vWF multimers, thereby favoring platelet-rich microvascular thrombosis and impairing tissue perfusion (101). This imbalance contributes to microvascular obstruction and the development of organ dysfunction (101, 102).

Importantly, immunothrombosis does not occur independently of other endothelial injury programs. Complement activation, cytokine signaling, oxidative stress, and glycocalyx disruption converge to enhance endothelial procoagulant activity and immune-cell interactions, creating a self-reinforcing thromboinflammatory environment. Thus, endothelial immunothrombosis represents a key convergence mechanism through which inflammation is translated into microvascular dysfunction and organ injury during sepsis.

## Organ-specific vulnerability of endothelial dysfunction

Although endothelial dysfunction occurs throughout the systemic circulation during sepsis, its clinical consequences vary among organs. This heterogeneity reflects differences in endothelial specialization, vascular architecture, tissue metabolic requirements, immune environment, and susceptibility to inflammatory and hemodynamic stress. Therefore, organ dysfunction in sepsis should be viewed not only as a consequence of systemic endothelial injury, but also as the result of organ-specific vascular responses to shared pathogenic mechanisms (Figure 2).

### Sepsis-induced acute lung injury and ARDS.

The lungs, among the most vulnerable organs during sepsis, are highly susceptible to barrier disruption and inflammatory injury. Pulmonary endothelial injury disrupts the alveolar-capillary barrier, promoting protein-rich edema formation, impaired gas exchange, and progression toward ARDS (103). Multiple endothelial injury programs contribute to pulmonary dysfunction, including glycocalyx degradation, junctional instability, inflammatory leukocyte recruitment, and oxidative stress. Activated endothelial cells exhibit increased adhesion molecule expression, neutrophil recruitment, and transendothelial migration into surrounding tissues, thereby further amplifying vascular permeability and inflammatory tissue injury (104, 105). In this context, pulmonary manifestations result from reduced barrier integrity with capillary leakage and inflammation. Other contributors to pulmonary endothelial injury during sepsis include microvascular thrombosis and impaired perfusion regulation, which promote ventilation-perfusion mismatch and persistent oxygenation abnormalities despite systemic hemodynamic stabilization (106, 107).

### Sepsis-induced AKI.

The kidneys are highly dependent on coordinated regulation of microvascular blood flow and endothelial cell–mediated filtration processes, making them susceptible to microcirculatory dysfunction and impaired filtration. Sepsis-associated AKI involves interactions among endothelial dysfunction, inflammation, altered microcirculation, and tubular injury (108). Unlike classical ischemic models of AKI, septic AKI may occur despite preserved or increased renal blood flow, indicating roles for endothelial dysfunction and microvascular dysregulation. Disrupted endothelial signaling, impaired vascular autoregulation, leukocyte-endothelial interactions, and microvascular inflammation contribute to heterogeneous perfusion abnormalities within the renal circulation (109, 110). Recent evidence in mice suggests that renal macrophages can limit endothelial IL-6 production through IL-1 receptor antagonist signaling, thereby attenuating inflammatory endothelial activation and kidney injury (111). Therefore, renal manifestations of endotheliopathy are characterized by impaired microvascular regulation, altered oxygen distribution, and loss of vascular-tubular homeostasis, rather than by global hypoperfusion. These findings emphasize that septic AKI arises from coordinated interactions among endothelial cells, immune populations, and tubular compartments rather than isolated renal ischemic injury.

### Sepsis-induced neurovascular dysfunction.

The central nervous system has a unique susceptibility to endothelial dysfunction due to the specialized properties of the neurovascular unit (112). Blood-brain barrier disruption and neuroinflammation are dominant endothelial phenotypes in sepsis. The blood-brain barrier depends on coordinated interactions among endothelial cells, pericytes, astrocytes, and extracellular matrix components to maintain neural homeostasis. During sepsis, inflammatory mediators, oxidative stress, and endothelial activation disrupt neurovascular integrity, leading to altered barrier permeability, immune-cell trafficking, and neuronal dysfunction (113, 114). These processes contribute to sepsis-associated encephalopathy, which is characterized by cognitive impairment, altered consciousness, and long-term neurological complications (115, 116). Although neurovascular dysfunction is an important manifestation of sepsis-associated encephalopathy, it should be interpreted within the broader context of systemic endothelial injury rather than as an isolated process. As with other organs, the severity of neurological manifestations depends on the balance between vascular injury, inflammatory amplification, and endogenous repair mechanisms.

### Sepsis-induced cardiovascular dysfunction.

The cardiovascular system represents a critical target of SAE, as endothelial integrity is essential for maintaining vascular tone, tissue perfusion, and cardiovascular homeostasis (117). The widespread distribution of endothelial cells across arterial, venous, and microvascular compartments makes the cardiovascular system particularly vulnerable to systemic inflammation, metabolic stress, and disturbances in endothelial signaling during sepsis (118). Unlike barrier-dominant organs such as the lung, in the cardiovascular system endothelial dysfunction is primarily characterized by impaired vascular regulation, altered microvascular perfusion, and loss of hemodynamic coherence. Restoration of blood pressure and cardiac output does not necessarily indicate recovery of microvascular function, as persistent endothelial dysregulation may impair regulation of vascular tone, microvascular flow distribution, and tissue oxygen delivery (119). Dysregulated NO signaling, inflammatory endothelial activation, and impaired vascular responsiveness contribute to vasoplegia and circulatory instability, representing important mechanisms underlying septic shock.

Beyond systemic vascular regulation, endothelial dysfunction within the coronary microcirculation may directly influence cardiac performance. Altered endothelial cell–cardiomyocyte communication, impaired coronary microvascular perfusion, and inflammatory vascular injury can contribute to myocardial oxygen imbalance, reduced cardiac efficiency, and the development of septic cardiomyopathy (120, 121). These changes suggest that cardiovascular dysfunction in sepsis is not solely attributable to direct myocardial injury but also reflects disruption of the vascular environment that regulates myocardial perfusion and metabolic adaptation. Therefore, cardiovascular endothelial dysfunction represents an organ-specific manifestation of SAE characterized by impaired vascular regulation, disrupted microvascular-cardiac interactions, and reduced cardiovascular adaptability to systemic stress (122).

## Diagnosis of endothelial dysfunction in sepsis

The clinical assessment of SAE is challenging because direct bedside measurement of endothelial function is rarely feasible. A combination of biomarkers and physiological indicators provides complementary information to diagnose endothelial activation, barrier disruption, microcirculatory dysfunction, and thromboinflammatory responses. Characterizing endothelial states and defining therapies for SAE requires an integrated interpretation of biological signals, bedside physiological findings, and their temporal changes (Figure 3).

Most circulating SAE biomarkers reflect downstream consequences of endothelial activation or injury and should therefore be interpreted within the context of the disease trajectory rather than as isolated indicators of specific endothelial states. Candidate biomarkers for sepsis-induced endothelial activation and dysfunction include signals of glycocalyx and barrier injury markers, barrier disruption, immunothrombosis risk, coagulopathy, and fluid intolerance. To facilitate interpretation of these biological signals within the phenotype- and trajectory-oriented framework, the representative mechanisms, biology-anchored biomarkers, bedside surrogates, major limitations, and potential translational implications associated with the four endothelial failure modes are summarized in Table 1.

Disruption of the endothelial glycocalyx is an early feature of SAE. Circulating components released during glycocalyx shedding, including syndecan-1, heparan sulfate, and hyaluronan, have been investigated as indicators of endothelial barrier injury and disease severity (73, 123, 124). Elevated syndecan-1 levels have been associated with increased vascular permeability, organ dysfunction, and adverse outcomes in patients with sepsis (125). However, these markers reflect glycocalyx degradation rather than directly indicating the functional consequences of barrier failure.

Markers of endothelial activation and permeability, including angiopoietin-2 (Ang-2), soluble thrombomodulin, vascular endothelial cadherin (VE-cadherin), and adhesion molecules such as ICAM-1 and VCAM-1, reflect different aspects of endothelial activation, junctional instability, and inflammatory endothelial signaling (126–129). Among these, Ang-2 has received particular attention because it reflects dysregulated endothelial activation and impaired Tie2 signaling, which contribute to vascular leakage and microvascular dysfunction (130).

Thromboinflammatory markers that are associated with endothelial coagulation activation include thrombomodulin, vWF, and the vWF-ADAMTS13 axis (102, 131). These markers may help identify patients with predominant thromboinflammatory endothelial states, although their clinical interpretation requires integration with platelet dynamics, coagulation profiles, and organ manifestations.

The integration of biomarker trajectories and functional assessment of vascular consequences provides complementary information about the status of the endothelium. Bedside indicators, including capillary refill time, skin mottling, lactate kinetics, and peripheral perfusion assessment, provide indirect evidence of microcirculatory dysfunction and impaired tissue oxygen delivery (22, 132–134). Advanced approaches, such as sublingual microcirculatory imaging, near-infrared spectroscopy, and dynamic evaluation of vascular responsiveness, further allow assessment of microvascular abnormalities that may persist despite normalization of conventional hemodynamic parameters (135–137).

These functional assessments are physiological manifestations of endothelial dysfunction, but they do not directly measure endothelial injury. Given the dynamic nature of SAE, integrating serial biomarker changes with bedside physiological trajectories may provide greater insight than single time-point measurements. For example, persistent microcirculatory abnormalities despite restored blood pressure, accompanied by progressive increases in glycocalyx injury markers, endothelial activation markers, or coagulation-related indicators, suggest a worsening state with more severe or mixed endothelial failure. Conversely, improvement in peripheral perfusion together with stabilization of endothelial cell–related biomarkers may indicate recovery of vascular homeostasis. Such longitudinal integration may provide a framework for recognizing evolving endothelial states and refining future approaches to risk assessment and therapeutic evaluation (Figure 3).

## Translational implications and future directions

The recognition of SAE as a dynamic, heterogeneous process has important implications for translational research and precision medicine. Current approaches to sepsis classification are primarily based on organ dysfunction and disease severity, but patients with similar clinical presentations may exhibit distinct endothelial states characterized by predominant barrier disruption, microcirculatory dysfunction, thromboinflammatory activation, or inflammatory amplification. An understanding of a patient’s endothelial state based on circulating biomarkers, functional assessments, and their clinical trajectory may provide a biological framework to refine patient prognosis and stratification and guide more targeted interventions.

The limited success of previous sepsis-directed therapies may partly reflect insufficient consideration of biological heterogeneity. Incorporating assessments of endothelial biomarkers and vascular function into clinical trial design to enrich for patient populations with specific endothelial abnormalities may improve the evaluation of targeted interventions. Several therapeutic strategies aimed at preserving glycocalyx integrity (138), restoring endothelial barrier function (139), modulating inflammatory signaling (140), or reducing thromboinflammatory activation (141) have been explored. However, most remain investigational, and their effectiveness may depend on a patient’s disease stage, endothelial trajectory, and the balance between adaptive and maladaptive vascular responses.

Despite advances in endothelial biology, several challenges remain before endotheliopathy-guided strategies can be translated into clinical practice. Standardized definitions of endothelial states, validated biomarker panels, and reliable approaches for longitudinal assessment are needed. Moreover, many circulating markers reflect downstream consequences of endothelial injury and require interpretation within broader biological and physiological contexts. Future studies integrating longitudinal multi-omics, advanced microcirculatory assessment, and clinical phenotyping may help define clinically meaningful endothelial trajectories and identify therapeutic windows. Ultimately, a more precise understanding of endothelial evolution may facilitate the transition from generalized sepsis management toward individualized vascular-informed strategies.

## Conclusions

The endothelium plays a central role in maintaining vascular homeostasis by preserving barrier integrity, regulating microvascular tone and flow distribution, coordinating hemostatic balance, and controlling leukocyte trafficking. In sepsis, diverse infectious and inflammatory stimuli converge on endothelial signaling pathways, driving a continuum from adaptive activation to dysfunction and structural injury. SAE is a dynamic, clinically heterogeneous process that extends beyond a single episode of endothelial injury. The endothelium integrates inflammatory, metabolic, immune, and hemostatic signals throughout the course of sepsis, resulting in evolving patterns of vascular dysfunction that contribute to barrier disruption, microcirculatory impairment, thromboinflammatory activation, and organ-specific injury.

The phenotype- and trajectory-oriented framework proposed in this Review emphasizes that clinically recognizable endothelial failure modes are not independent biological subtypes but overlapping manifestations of shared molecular programs whose relative dominance changes over time. Understanding these evolving endothelial states provides a conceptual link between molecular mechanisms, bedside manifestations, and organ-specific vulnerability.

Future advances will require integration of biology-anchored biomarkers, functional vascular assessments, and longitudinal trajectory analysis to better characterize endothelial heterogeneity in sepsis. Although substantial challenges remain, a more precise understanding of endothelial evolution may facilitate the transition toward individualized, vascular-informed approaches for sepsis management.

## AI disclosure

During the preparation of this manuscript, Grammarly (Grammarly Desktop Version 1.192.0, date range July 26 to August 2, 2026) and ChatGPT (ChatGPT Version 5, date ranges January 20–25, March 20–23, April 9–11, July 28–30, 2026) were used only for English-language editing and grammatical refinement, not for generating scientific content. The authors reviewed all suggested changes and take full responsibility for the manuscript.

## Conflict of interest

The authors have declared that no conflict of interest exists.

## Funding support

- UCSF Department of Anesthesia and Perioperative Care (to JH, FX, and SQ).
- Foundation for Anesthesia Education and Research: Medical Student Anesthesia Research Fellowship (to CZ).
- National Natural Science Foundation of China (grant 82460373 to SQ).

## Figures and Tables

**Figure 1:**
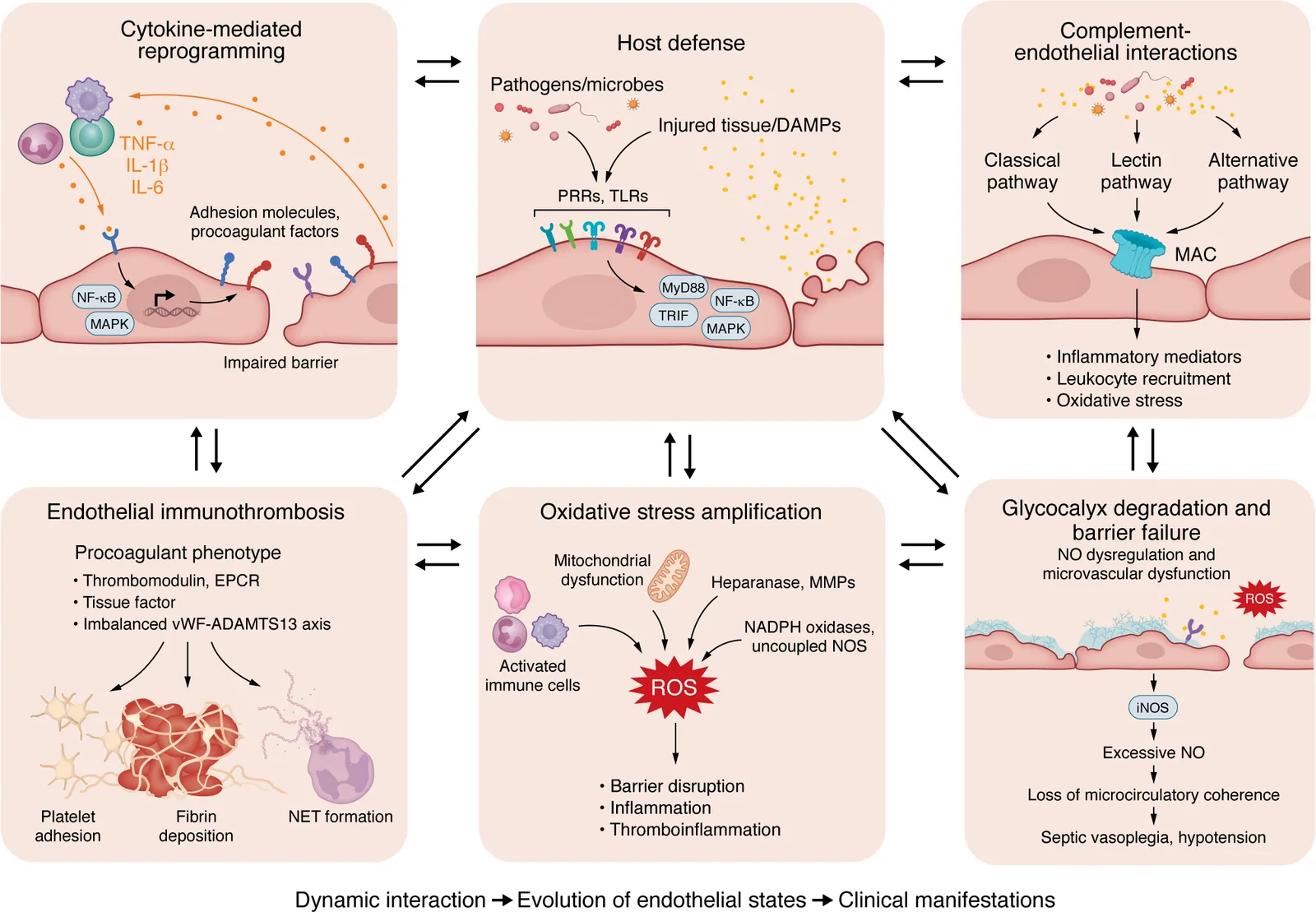
Molecular mechanisms and manifestations of sepsis-induced endothelial dysfunction. This diagram illustrates the interplay of molecular programs during sepsis. Components of pathogens (PAMPs) and injured tissues (DAMPs) activate endothelial pattern recognition receptors (PRRs), such as the TLRs. During sepsis, initial host defenses give way to excessive activation and interconnected pathological programs: cytokine signaling, complement system activation, immunothrombosis, and glycocalyx degradation, leading to barrier failure, NO dysregulation, and oxidative stress, which acts as a central amplifier of dysfunction. These processes interact dynamically, determining endothelial state transitions and their clinical manifestations.

**Figure 2:**
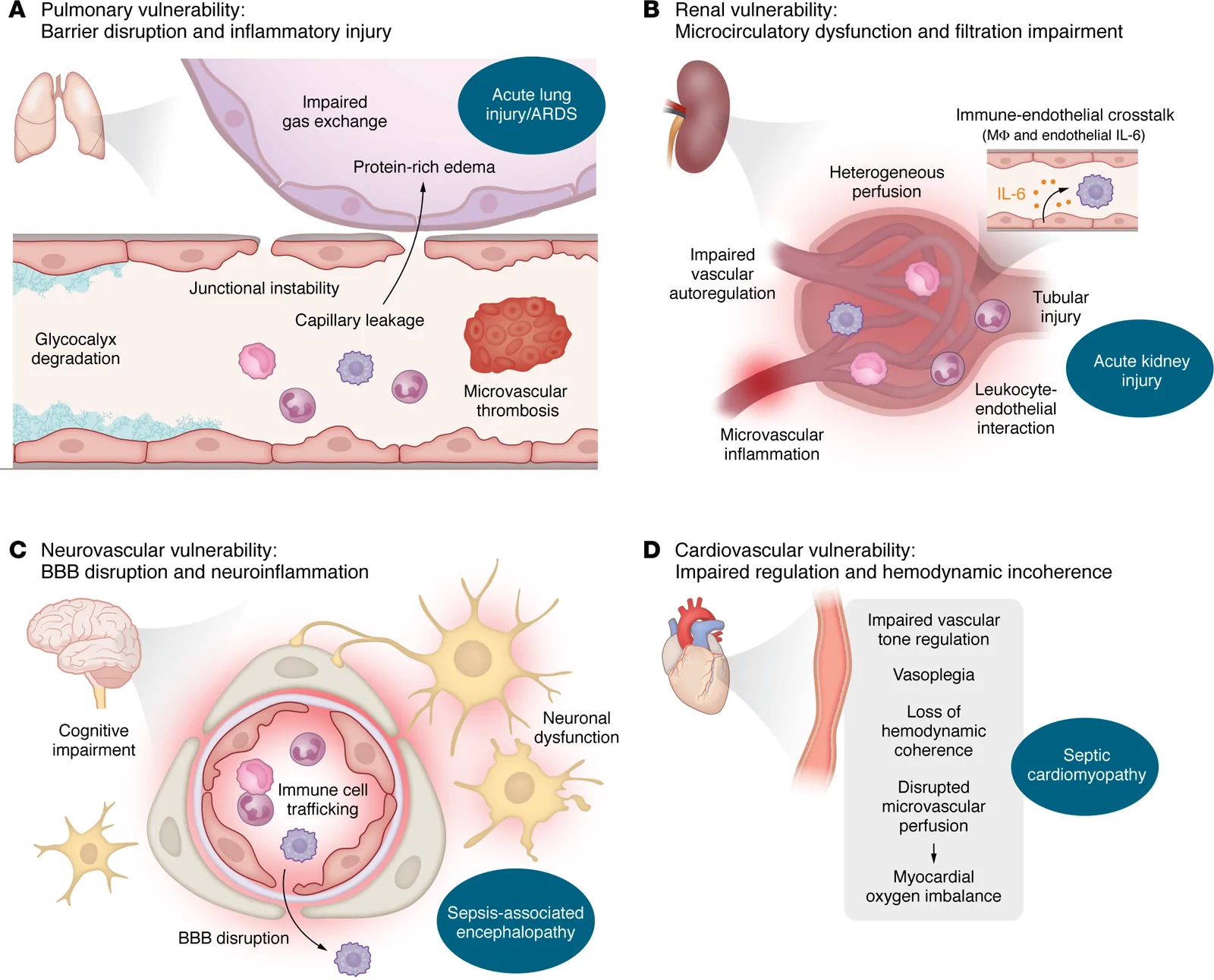
Organ-specific manifestations of endothelial dysfunction in sepsis. The schematic overview illustrates the heterogeneous pathological responses of the endothelium across different organ systems during sepsis. (**A**) Pulmonary vulnerability is characterized by glycocalyx degradation and junctional instability leading to capillary leakage, protein-rich edema, microvascular thrombosis, and impaired gas exchange (acute lung injury and ARDS). (**B**) Renal vulnerability involves microvascular inflammation, impaired autoregulation, and heterogeneous perfusion, which drive tubular injury; this is moderated by immune-endothelial crosstalk, e.g., between macrophages and endothelial IL-6. (**C**) Neurovascular vulnerability manifests as disruption of the blood-brain barrier (BBB) within the neurovascular unit, leading to immune cell trafficking, neuronal dysfunction, and cognitive impairment (sepsis-associated encephalopathy). (**D**) Cardiovascular vulnerability is characterized by impaired regulation of vascular tone (vasoplegia), loss of hemodynamic coherence between macro- and microcirculation, and disrupted coronary microvascular perfusion, resulting in myocardial oxygen imbalance (septic cardiomyopathy).

**Figure 3:**
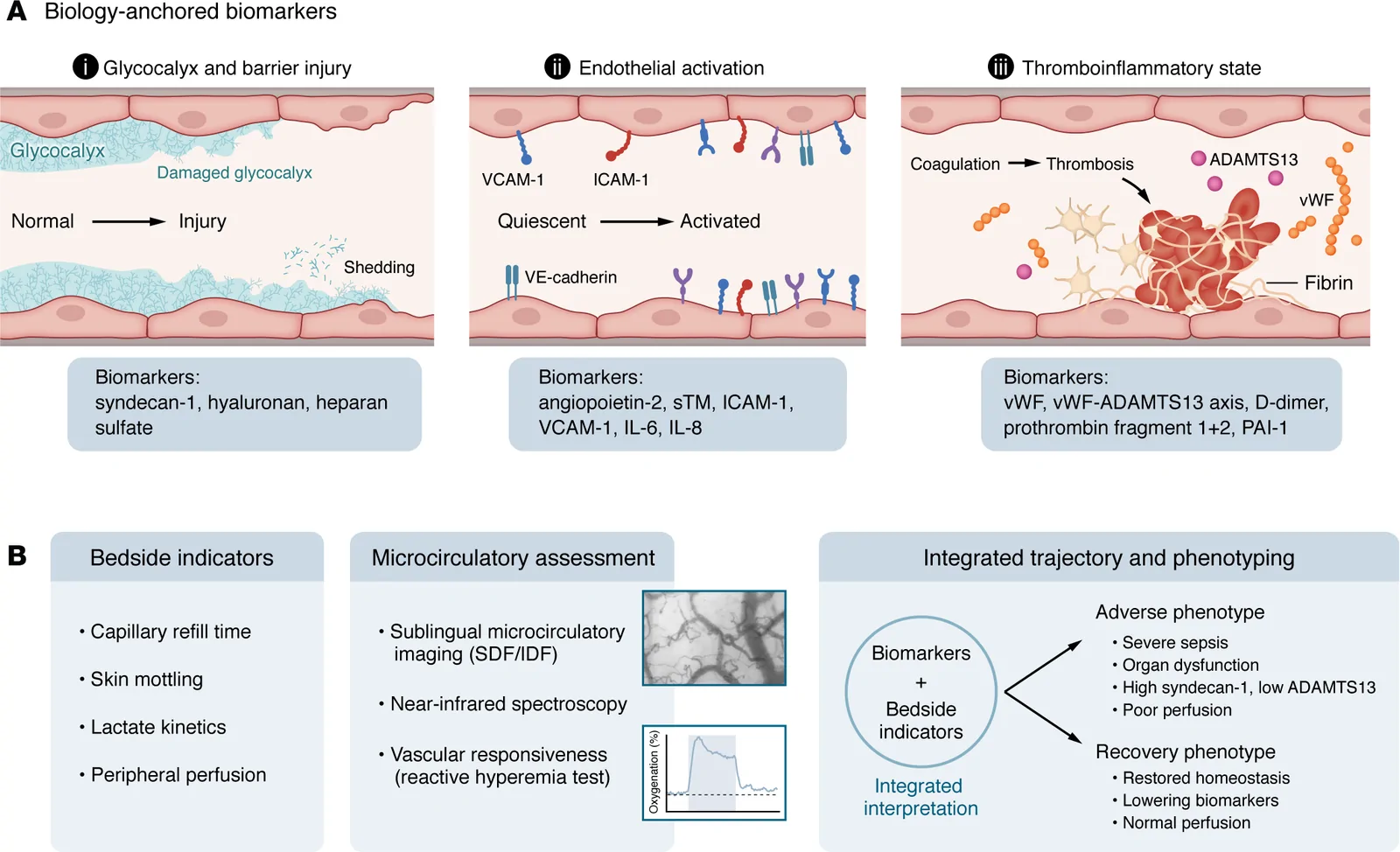
Biomarkers and integrated bedside assessment for sepsis-associated endotheliopathy. (**A**) Mechanisms and corresponding biology-anchored biomarkers categorized into three key pathological processes: (i) glycocalyx and barrier injury (e.g., syndecan-1, hyaluronan, heparan sulfate), (ii) endothelial activation (e.g., Ang-2, ICAM-1, IL-6, IL-8), and (iii) thromboinflammation (e.g., vWF, ADAMTS13, PAI-1). (**B**) Complementary bedside assessment strategies, including conventional microcirculatory indicators, advanced microcirculatory imaging (sidestream dark field [SDF]; incident dark field [IDF]), and functional tests (i.e., near-infrared spectroscopy), are integrated over time. The temporal trajectory, which integrates biomarker dynamics with physiological status, informs the distinction between an adverse and a recovery phenotype.

**Table 1:**
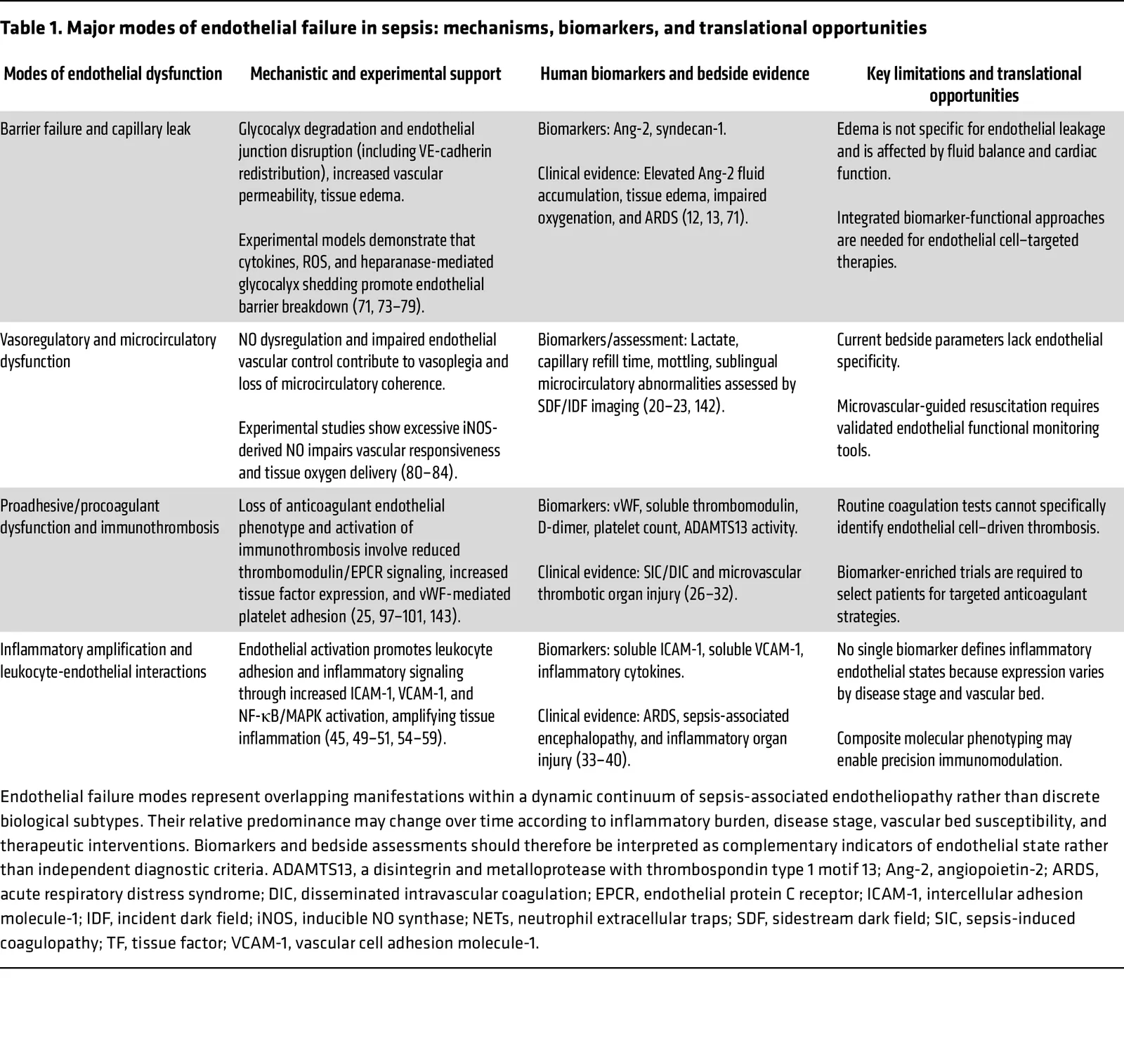
Major modes of endothelial failure in sepsis: mechanisms, biomarkers, and translational opportunities

## Version 1 10/15/2026

Electronic publication
